# Rapid diagnostic value of next-generation sequencing-based technologies in childhood pneumonia

**DOI:** 10.3389/fped.2025.1662367

**Published:** 2026-01-20

**Authors:** Yanfei Chen, Xiaoli Zhu, Fang Fang, Kaihui Ma, Yanli Zhang, Hongxia Liu

**Affiliations:** 1 Department of Clinical Laboratory, Yancheng Maternal and Child Health Care Hospital Affiliated to Yangzhou University, Yancheng, Jiangsu, China; 2 Department of Clinical Laboratory, The Affiliated Taizhou People’s Hospital of Nanjing Medical University, Taizhou, Jiangsu, China

**Keywords:** pneumonia, pneumococcus pneumoniae, polymerase chain reaction, blood, diagnosis

## Abstract

**Background:**

This study evaluates the diagnostic efficacy of next-generation sequencing (NGS) in pediatric patients with suspected pneumonia and unidentified etiologies.

**Objective:**

This retrospective study encompassed pediatric patients with suspected pneumonia, spanning the period from January 2022 to December 2023. Nasal swabs and blood samples were collected for a comprehensive diagnostic panel, including NGS, blood culture, complete blood count, and serum biomarkers.

**Methods:**

Routine diagnostic tests were compared with NGS for turnaround time and diagnostic accuracy. Patients were categorized based on clinical diagnosis into non-pneumonia and pneumonia groups. Logistic regression analysis was performed to identify independent predictors of pneumonia.

**Results:**

NGS provided results within 24 h, significantly faster than conventional bacterial cultures (3–5 days). The positivity rate for pathogen identification increased from 55.3% with traditional methods to 86.2% with NGS (*p* < 0.05). Serum levels of procalcitonin, creatinine, and C-reactive protein were elevated in pneumonia patients, while albumin levels were decreased. Logistic regression identified C-reactive protein and albumin as independent predictors of pneumonia. The area under the receiver operating characteristic curve for NGS was superior to conventional methods and serum biomarkers alone or in combination.

**Conclusion:**

NGS is a promising tool for rapid and accurate etiologic diagnosis of pneumonia in children. The combination of NGS with albumin levels may serve as an effective screening strategy, potentially enhancing clinical management through earlier intervention and targeted therapy. Further validation in larger cohorts is warranted to establish the clinical utility of this approach.

## Introduction

According to the World Health Organization, pneumonia is a significant category of disease that poses a severe threat to children's health and continues to be one of the leading causes of mortality among children under the age of five worldwide (1). Particularly, severe pneumonia, characterized by its rapid progression, high morbidity and mortality rates, and potential to leave lasting respiratory complications, necessitates early detection and prompt treatment (1). The limited sensitivity of current diagnostic methods poses significant challenges in diagnosing childhood pneumonia. Traditional approaches to identifying the etiology of pneumonia in children, such as blood cultures, often exhibit low sensitivity, particularly for detecting the causative organisms of bacterial pneumonia (2, 3). Therefore identifying the causative agent of an infectious disease is very important, and traditional microbiological tests have many disadvantages, such as the time required, the small number of types of tests, and the low detection rate of pathogens (4). The early identification of pathogens primarily depends on bacterial culture and polymerase chain reaction (PCR) techniques. However, these methods often fall short in meeting the actual detection requirements due to limitations in specificity and sensitivity (2–4). Therefore, rapid and accurate diagnosis of pathogens plays an important role in the early treatment and recovery of patients.

The development of tools to diagnose pneumonia pathogens has been slow. Because of the close homology of Streptococcus, many of the causative organisms that cause pneumonia, some early genetic PCR tests have been found to be non-specific for the diagnosis of *Streptococcus pneumoniae* as well (5–9). As a result, the clinical specificity of tests for pneumococcal pathogens has been difficult to measure, particularly in children, who are the population most in need of improved diagnostic testing. In healthy children, either no or extremely low concentrations of pathogenic bacteria were detected as positive, indicating that the test achieves 100% specificity. However, when it comes to diagnostic sensitivity and specificity, our findings reveal that the accuracy of the false-positive rate is lower among healthy patients (4, 10). Other studies have also found that the higher the concentration and variety of pathogenic bacteria in patient samples, the more severe the condition, so that children with pneumonia may have higher levels of pathogenic bacteria in the respiratory mucosa than healthy children (4–7).

Next-generation sequencing (NGS) is an all-encompassing assay technology that can detect a wide range of pathogens. Several studies in recent years have demonstrated the unique advantages and feasibility of NGS in identifying pathogens of respiratory tract infections. Therefore, in our study, we evaluated the efficacy of NGS as a diagnostic test for pathogenic organisms in patients with pneumonia and analysed the associations between pathogenic microorganisms of pneumonia and markers of clinical characteristics in children, in order to facilitate the proposal of a new and more accurate and rapid diagnostic combination method.

## Methods

### Research design

**Figure 1:**
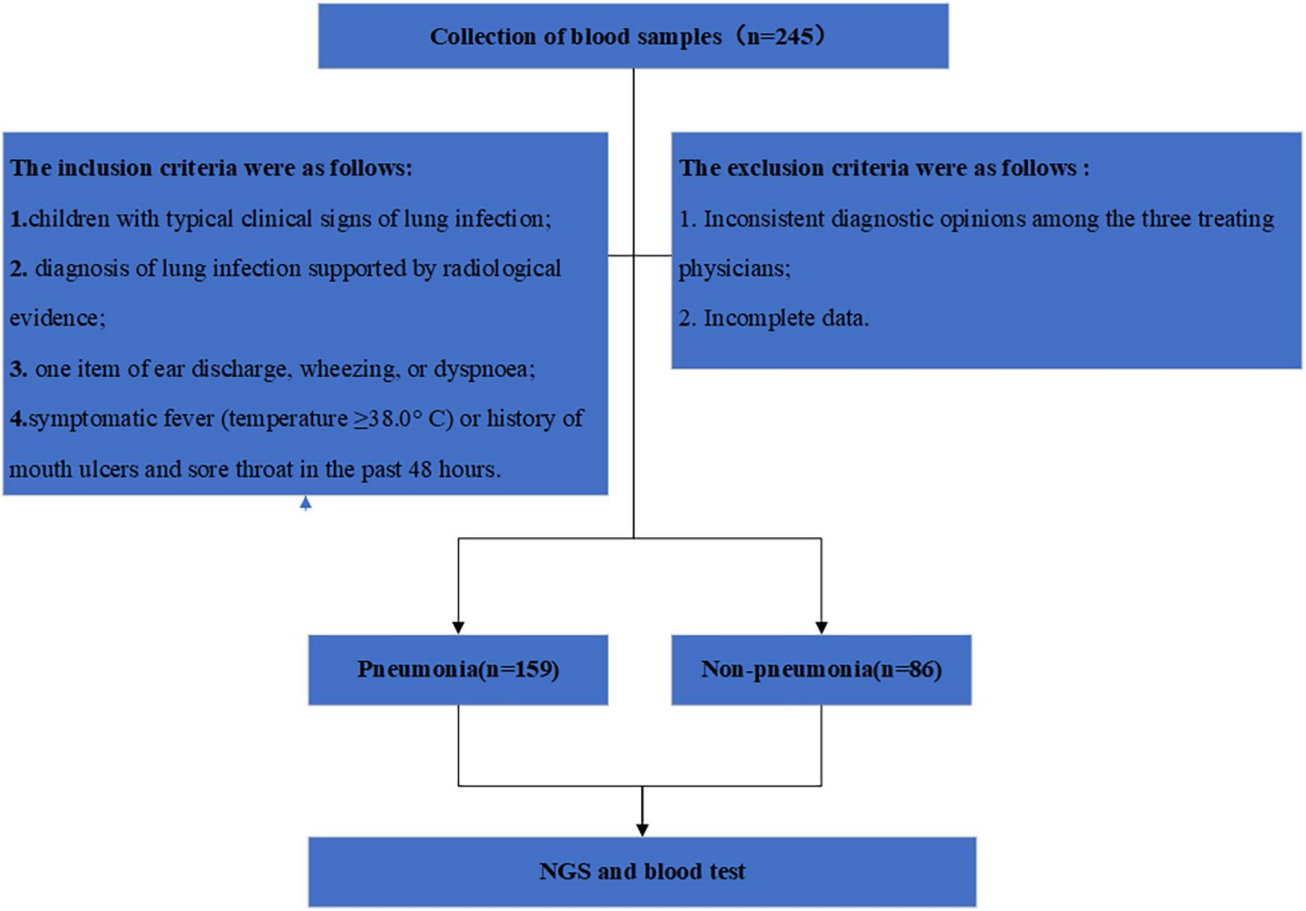
Research workflow diagram.

This is a retrospective observational study with a sample of paediatric pneumonia patients from Yancheng Maternal and Child Health Hospital affiliated to Yangzhou University and Taizhou People's Hospital affiliated to Nanjing Medical University from January 2020 to December 2023 (Figure 1). The etiology of pneumonia was assessed in hospital and registered for 24 consecutive months. Cases were children aged 0–12 years who were hospitalised for pneumonia as defined by the World Health Organisation (WHO). Controls were randomly selected from the community and frequency matched to cases for age (1–<6 months, 6–6 months). Both cases and controls were assessed for clinical signs and symptoms and risk factors for pneumonia at the time of enrolment. Inclusion criteria were as follows (1) children with typical clinical signs of lung infection, such as fever, cough, sputum, and dyspnea, and (2) diagnosis of lung infection supported by radiological evidence (chest computed tomography). Chest x-ray (CXR) performed at the time of admission, where respiratory tract illness (RTI) was defined as cough or runny nose in controls. RTI was also defined as: (1) at least one item of ear discharge, wheezing, or dyspnoea; and (2) symptomatic fever (temperature ≥38.0 °C) or history of mouth ulcers and sore throat in the past 48 h. The exclusion criteria were as follows: (1) inconsistent diagnostic opinions among the three treating physicians; (2) incomplete data. At all sites, nasal swab samples from cases and controls were collected along with routine blood tests, and nasopharyngeal swab samples were tested for both NGS and bacterial culture, this work was approved by the Ethics Committee of Yancheng Maternal and Child Health Hospital Affiliated to Yangzhou University (2023-LW-008).

### Traditional microbiological testing

Nasal swabs of patients were collected within 1–2 days of admission for routine microbiological testing, including microbiological culture, nasal swab multiplex PCR and antigen detection testing.

### Clinical diagnostic criteria

In the context of clinical diagnostics, the examination of both traditional microbiological assays and NGS data, alongside a thorough review of patients' medical charts, is essential for accurate disease identification and management. In this scenario, two seasoned clinicians conducted a detailed evaluation to ascertain the presence of pneumonia in each case.

Initially, the clinicians employed a systematic approach to diagnose pneumonia by considering a range of clinical parameters. These parameters included observable symptoms, radiographic findings in lung imaging, and results from laboratory tests consistent with the condition. Their diagnostic process strictly adhered to the “Diagnostic Guidelines for Pneumonia in Chinese Children,” as outlined by the National Health Commission of the People's Republic of China and the State Administration of Traditional Chinese Medicine in their 2019 guidelines. Once pneumonia was diagnosed, the clinicians moved on to identify the etiology of the disease. This involved a comprehensive analysis of each patient's clinical presentation, medical history, and the results from both conventional microbiological assays and NGS. The microbiological assays facilitated the detection of potential infectious agents, while NGS provided a more in-depth understanding of the genetic makeup of the pathogens, if any, involved in the disease process.

By integrating the wealth of information obtained from these various sources, the clinicians were able to determine the underlying cause of pneumonia in each patient. This multifaceted diagnostic approach ensures that the most appropriate treatment strategies are implemented, thereby optimizing patient outcomes and contributing to the overall advancement of medical practice within the framework of evidence-based medicine.

### Nucleic acid extraction, library construction and sequencing work

Bronchoscopy was conducted utilizing a flexible fiberoptic endoscope in accordance with established protocols, with specimens being preserved at a temperature of 4 °C. Nasal swab samples were submitted for NGS analysis, encompassing both deoxyribonucleic acid (DNA) and ribonucleic acid (RNA) assessments. DNA was isolated employing a DNA extraction kit (Tiangen), adhering to the guidelines provided by the manufacturer, and environmental DNA was eliminated through the application of enzymatic DNA degradation. Total RNA was extracted using an RNA isolation kit (Tiangen). Ribosomal RNA (rRNA) was excised utilizing an rRNA removal kit (Omega). Ribosomal RNA was transcribed using reverse transcriptase and deoxyribonucleoside triphosphates. Complementary DNA (cDNA) was synthesized leveraging reverse transcriptase and deoxynucleoside triphosphates (Thermo Fisher Scientific). Both DNA and cDNA libraries were assembled through the employment of the DNA Library Preparation Kit (Omega). The quality of the libraries was appraised using the dsDNA HS Detection Kit (Omega), followed by the High Sensitivity DNA Kit (Omega), on an Agilent 2100 Bioanalyser. Subsequently, the library pools were loaded onto an Illumina NextSeq CN500 sequencer to undergo 75 cycles of single-ended sequencing, yielding an approximate total of 20 million reads per library. To establish negative controls, nasal swab samples sourced from healthy individuals were analyzed following identical protocols. Sterile deionized water was extracted alongside the specimens to serve as a non-template control.

### Sequence Reading and analysis

To enhance the quality of sequence data, reads of low quality, duplicate reads, and those shorter than 50 base pairs (bp) were culled from the dataset. Additionally, reads exhibiting low complexity were discarded employing default parameter settings. Human-derived sequence data were pinpointed and subsequently excluded by mapping them against the human reference genome (hg38) using the Burrows-Wheeler Aligner software tool. A curated selection of representative microbial sequences, including bacteria, viruses, fungi, and other eukaryotic pathogens, was obtained from the NCBI Nucleotide and Genome Database, which is managed by the National Centre for Biotechnology Information (NCBI).

This selection was informed by the authoritative “Handbook of Clinical Microbiology” and corroborated by case reports and research studies published in contemporary, peer-reviewed scientific journals (10, 11).The resultant comprehensive database encompasses approximately 13,000 distinct microbial genomes. The microbial sequence reads were then aligned to this refined database using the SNAP v1.0 beta 18 software (12). A positive viral identification was ascertained when three or more non-overlapping genomic regions demonstrated adequate coverage. A species or genus was deemed positive for presence in a clinical sample when the Relative Microbial Proportion (RMP) value reached a threshold of 25. The RMP is calculated by dividing the Relative Proportion Measure (RPM) observed in the clinical sample by the RPM detected in the negative control specimen (13). This method ensures a robust and reliable interpretation of the sequencing data, facilitating accurate identification of microbial agents in clinical samples.

### Statistical analysis

IBM SPSS Statistics 19.0 was utilized for data analysis. Diagnostic performance was benchmarked against comprehensive clinical and microbiological assessments. Sensitivity, specificity, positive predictive value (PPV), negative predictive value (NPV), and accuracy were calculated for pathogen-level diagnostics using standard formulas, with 95% confidence intervals (CIs) determined via Wilson's method. McNemar's test compared the diagnostic efficacy of conventional microbiology with NGS, with two-tailed tests and a *p*-value <0.05 indicating statistical significance. The study reported these metrics to enable direct comparison between NGS and traditional methods, noting the presence of multiple infections in some pediatric cases.

## Results

### General characteristics of the patient

In this investigation, a cohort of 245 subjects was stratified into two distinct groups predicated on clinical ascertainment: individuals afflicted with pneumonia (64.9%, 159/245) and those in a state of health (35.1%, 86/245). A cohort of 86 ostensibly healthy pediatric subjects were utilized as a comparator group, and a total of 159 patients fulfilled the predefined eligibility criteria, comprising 84 males and 75 females, with an age range spanning from one month to 13 years. Inclusion of pneumonia patients and healthy control subjects was predicated on dual diagnostic modalities, namely conventional microbiological identification and NGS (Table 1). Serum concentrations of creatinine (CREA), procalcitonin (PCT), and C-reactive protein (CRP) were observed to be elevated in the pneumonia cohort in comparison to the healthy control group, while levels of albumin (ALB), urea, and erythrocyte counts were found to be diminished in the pneumonia cohort (*p* < 0.05). No statistically significant disparities (*p* > 0.05) were noted with respect to age, gender, leukocyte counts, and the incidence of positive blood culture results between the two cohorts (Table 1).

**Table 1:** Comparison of demographic and diagnostic characteristics between two groups [*n* (%)].

Variables	Pneumonia		*z*-Value	*p*-Value
	Yes (*n* = 159)	No (*n* = 86)		
Sex (M/F)	84 (52.83)/75 (47.17)	68 (79.07)/18 (20.93)	0.846	0.405
BC (+/−)	34/85 (40.00)	—	—	0.024
NGS (+/−)	72/85 (84.70)	—	—	0.007
PCT (μg/L)	2.12 (0.58, 4.33)	1.46 (0.21,3.15)	−2.116	0.514
ALB (g/L)	29.37 ± 4.55	36.35 ± 4.33	−3.213	0.005
CREA (μmol/L)	138.05 (65.39, 174.09)	164.25 (54.36, 198.15)	−2.283	0.606
WBC (×10^9^/L)	11.74 (7.16, 14.18)	12.35 (8.14,24.52)	−0.207	0.705
CRP (mg/L)	125.01 (35.77, 243.06)	46.29 (12.64,155.15)	−2.244	0.004

BC, bacterial culture; NGS, next generation sequencing; PCT, procalcitonin; ALB, Albumin; CREA, creatinine; WBC, leucocyte; CRP, C-reactive protein.

### Comparison and consistency of NGS with traditional microbial identification

**Figure 2:**
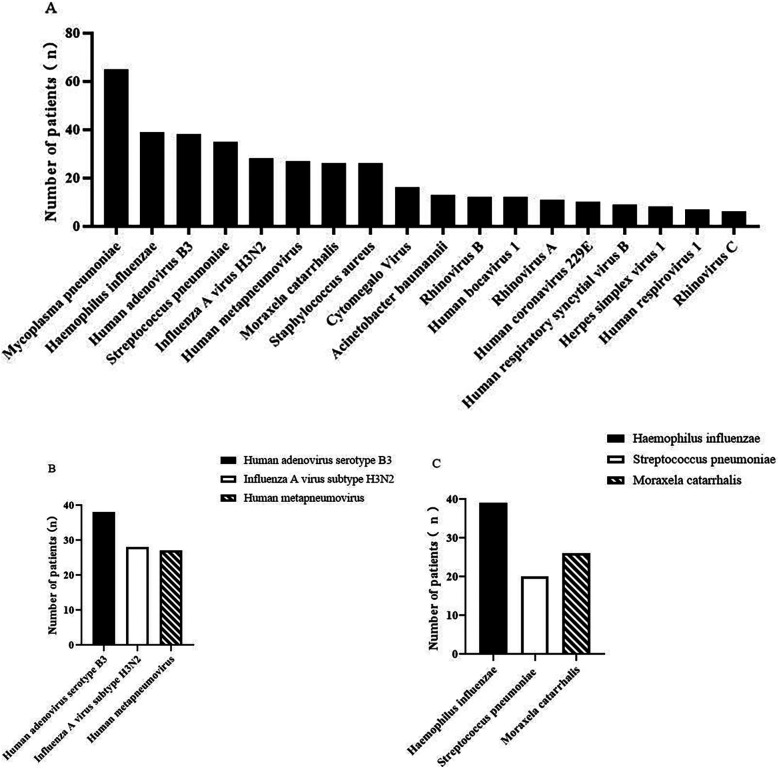
Distribution of pathogenic bacteria in patients with pneumonia. Distribution of the main pathogenic microorganisms in patients with pneumonia **(A)**, where **B** is the distribution of the main pathogenic viruses and **C** is the distribution of the main pathogenic bacteria.

Microbiological profiling of the patient population revealed that 55.3% (88/159) tested positive using traditional diagnostic techniques, whereas an increased proportion of 86.2% (137/159) exhibited positive results through NGS, indicating a statistically significant difference (*p* < 0.05). An extensive clinical evaluation identified a specific etiological agent in 86.2% (137/159) of the cases. Figure 2 delineates the prevalence of infectious agents that satisfied the established diagnostic criteria for infection. *Human adenovirus serotype B3* (38 cases), *Influenza A virus subtype H3N2* (28 cases), and *Human metapneumovirus* (27 cases) were identified as the most prevalent viral pneumonia infections. In the bacterial etiology, *Haemophilus influenzae* (39 cases), *Streptococcus pneumoniae* (20 cases), and *Moraxela catarrhalis* (26 cases) were the leading causes of bacterial pneumonia. Additionally, *Streptococcus pneumoniae* and *Mycoplasma pneumoniae* were the predominant atypical pathogens implicated in the study population. In addition, NGS has the advantage of a shorter diagnostic time. The average identification time for bacterial blood cultures is 3–5 days, whereas NGS identifies in as little as 24 h.

Table 2 delineates the prevalence of monomicrobial and polymicrobial infections, with 74 cases (46.5%) exhibiting monomicrobial infections and 85 instances (53.4%) of polymicrobial infections, which were further categorized into 53 double, 23 triple, and 9 quadruple microbial co-infections. In the cohort with monomicrobial infections, 34 of the 85 polymicrobial infections were identified using conventional diagnostic methodologies, representing 40% of the polymicrobial group (34/85). In contrast, NGS detected 72 of the polymicrobial infections, accounting for 84.7% of this subgroup (72/85). For both monomicrobial and polymicrobial infection scenarios, the detection efficacy of NGS was markedly superior to that of traditional diagnostic techniques, as indicated by a statistically significant difference (*p* < 0.05). The predominant pattern of co-infection involved a combination of bacterial and viral pathogens.

**Table 2:** Logistic regression analysis of sepsis.

Variables	B	Wald	*p*-value	OR	95% CI
NGS	−5.715	13.135	0.001<	0.004	0.000–0.045
ALB (g/L)	−0.224	4.228	0.021	0.855	0.674–0.916
CREA (μmol/L)	0.014	0.873	0.374	1.112	0.981–1.050
PCT (μg/L)	0.138	0.896	0.350	1.254	0.876–1.354
CRP (mg/L)	0.006	1.115	0.035	1.213	0.995–1.028

BC, bacterial culture; NGS, next generation sequencing; PCT, procalcitonin; ALB, Albumin; CREA, creatinine; WBC, leucocyte; CRP, C-reactive protein.

### Comparison of the diagnostic performance of traditional microbiological identification methods with NGS

**Figure 3:**
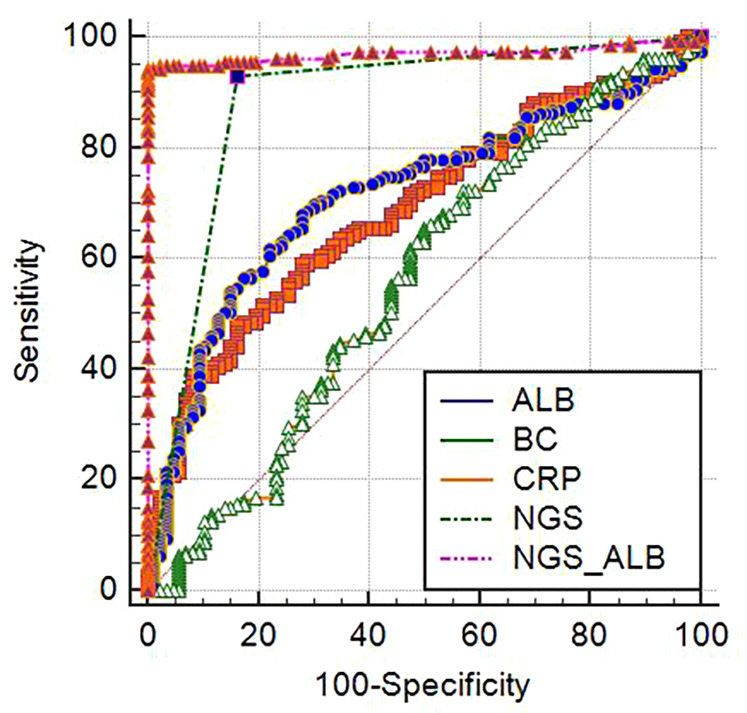
ROC curves of different indexes. *BC, bacterial culture; NGS, next generation sequencing; PCT, procalcitonin; ALB, Albumin; CREA, creatinine; WBC, leucocyte; CRP, C-reactive protein.

The diagnosis of clinical pneumonia was used as the “final criterion”. The area under the ROC curve based on routine microbiological identification (bacterial culture, BC) was 0.715 (95% CI: 0.654–0.771), with a sensitivity of 67.92% and a specificity of 72.09% (Figure 3), the area under the ROC curve based on routine blood CRP levels was 0.560 (95% CI: 0.495–0.623), with a sensitivity of 65.41% and a specificity of 50.00%, the area under the ROC curve (AUC) based on routine blood ALB levels was 0.686 (95% CI: 0.624–0.774), with a sensitivity of 47.8% and a specificity of 83.72%. The AUC of NGS was 0.884 (95% CI: 0.837–0.921), with a sensitivity of 93.08% and a specificity of 83.72%. The AUC of NGS + ALB was 0.972 (95% CI: 0.942–0.989), with a sensitivity of 94.34% and a specificity of 100%, the AUC was significantly higher than that of NGS alone (Figure 3).

### Multi-factor logistic regression analysis

After multifactorial logistic regression analysis C-reactive protein and ALB were found to be independent factors for pneumonia (Table 2).

## Discussion

A minority of pneumonia patients yielded positive results in traditional microbiological assays, despite the prevalence of potential bacterial infections being significant (14). This discrepancy can be attributed in part to technical limitations associated with sample collection and lesion localization in conventional diagnostic procedures, stringent conditions required for testing, and the generally low concentration of viable bacteria in clinical samples. Pneumonia, a grave infectious condition, poses a substantial risk of progressing to septic shock and potentially culminating in mortality (15, 16). A plethora of research has consistently indicated that expeditious and prompt identification of causative agents in pneumonia is paramount for prognostic outcomes, although the findings are often heterogeneous and multifaceted (17, 18). The initial clinical assessment of pneumonia by medical practitioners predominantly relies on the evaluation of inflammatory biomarkers and etiological tests, such as microbiological cultures, CRP, PCT, WBC count, and other indicators of inflammation. The precision and velocity with which a pathogenic etiology is determined are of paramount importance to intensivists, significantly influencing the selection of therapeutic strategies and, consequently, the prognosis of the patient. The accuracy and swiftness of these assessments are critical in the intensive care unit (ICU) setting, where timely and appropriate interventions can be the difference between life and death.

In alignment with prior research (19, 20), CRP and PCT, which are cost-effective clinical markers for inflammation, were observed to be markedly elevated in individuals with pneumonia compared to the healthy controls. Typically, PCT and CRP levels are maintained within a normal range in patients without inflammation. However, a systemic inflammatory response, such as that seen in pneumonia, can disrupt this balance and lead to not only increased levels of these biomarkers but also deranged metabolic functions.

The development of shock in pneumonia, a severe complication, extends beyond respiratory impairment, affecting the circulatory and neurological systems and, in extreme cases, leading to peripheral circulatory failure, which can be life-threatening (21). Our findings corroborate this understanding. Nevertheless, it is essential to recognize that CREA, CRP, and PCT, as non-specific biomarkers, can be influenced by various factors (22). The logistic regression analysis in this study revealed that PCT, CRP, and CREA did not emerge as independent risk factors for sepsis (*p* > 0.05), which is in concordance with numerous previous studies. The presence of confounding elements necessitates further investigation into their role as standalone predictors of pneumonia. A serum PCT level of 2.2 ng/mL is often indicative of inflammation. In the context of severe infections stemming from pathogenic infections, particularly those caused by gram-negative bacteria, both PCT and CRP levels escalate rapidly. Consequently, these biomarkers can serve as preliminary indicators of infection for pneumonia diagnosis. Furthermore, the progressive increment in CRP and PCT values more accurately mirrors the escalation of inflammation within the body than traditional indicators such as white blood cell count and body temperature. Notably, these results are typically available within an hour of admission. Prior studies have suggested that CREA, CRP, and PCT may aid in the diagnosis of severe pulmonary inflammation (23). This assertion is further supported by Zhang et al. (24).

In addition to PCT and CRP, ALB, recognized as a pro-inflammatory molecule, warrants increased scrutiny (25). The interplay between pneumonia and ALB has garnered significant interest among researchers (26). A robust correlation exists between ALB and inflammatory mediators (27). In severe pneumonia, plasma levels of IL-10, lipotoxins, and various soluble and protective proteins are substantially diminished (28, 29). Concurrently, heightened vascular permeability results in the shifting distribution of ALB between intravascular and extravascular spaces (30). Our study observed reduced ALB levels in the group with inflammatory lung disease compared to the healthy controls, with logistic regression suggesting a correlation between decreased albumin levels and an elevated risk of pneumonia. This correlation implies that diminished ALB concentrations may serve as a marker for pulmonary inflammation. Consequently, albumin levels should be closely monitored upon admission in suspected cases of lung infection.

In recent years, NGS technology has seen significant advancements, becoming more mature and widely integrated into clinical practice (31). The technology offers several benefits, such as rapid turnaround time, with results often available within 24 h (32), a broad range of microbial profiling capabilities (33), and the ability to perform semi-quantitative analyses (34). These advantages have established NGS as a powerful diagnostic tool in clinical settings. The efficiency of NGS is notable as it can directly detect thousands of microbial fragments in a single test, reducing the number of required tests and the time needed for assay (35). Moreover, NGS is less influenced by the presence of antibiotics and the activity of the pathogen, providing a clear advantage in pathogen identification. In contrast, traditional identification methods, while considered the gold standard, are often limited by their longer turnaround times and lower sensitivity, which can restrict their clinical application. In the current study, the benefits of NGS were evident, with a significant difference in the detection rates between traditional methods and NGS. Specifically, 55.3% (88/159) of the cases tested positive by traditional methods, while 86.2% (137/159) tested positive by NGS, indicating a statistically significant difference (*p* < 0.05) (30). This disparity may be attributed to several factors, including the fact that many patients had been treated with broad-spectrum antibiotics prior to sampling, which can complicate the collection and identification of pathogens. Additionally, the retention of pathogenic microorganism DNA in the nasal mucosa of patients for extended periods may also contribute to the higher detection rates observed with NGS.

Furthermore, NGS has the advantage of providing faster results compared to microbial cultures, whose average feedback time is typically 3–5 days (29). This rapid identification is particularly beneficial in the context of severe pneumonia, where timely diagnosis can significantly impact patient outcomes. The current study also highlighted the limitations of traditional methods in diagnosing viral infections. While molecular analyses, such as PCR-based methods, are established for clinical detection, they are often limited to known pathogens listed on the panel. In contrast, NGS identified a variety of viruses, including Human adenovirus B3, Influenza A virus H3N2, and Human metapneumovirus, which are not typically detected by conventional methods (30). This finding underscores the importance of NGS in identifying a broader spectrum of pathogens, including those that may be overlooked by traditional approaches.

NGS represents a significant advancement in pathogen identification, capable of detecting a wide range of pathogens, including viruses, bacteria, and fungi. This comprehensive detection is a marked improvement over conventional methods, which typically identify only single pathogens (30). NGS can identify multiple pathogenic bacteria and diagnose mixed infections, which is particularly valuable in complex cases where growth conditions are challenging or where competition between different pathogen types complicates diagnosis. In the context of mycotic infections, NGS has revolutionized diagnosis and management. It allows for direct and accurate identification of fungal species from clinical samples, which is especially advantageous for difficult-to-culture fungi like Pneumocystis jirovecii (36). Rapid diagnosis is crucial for timely treatment, especially in invasive infections. NGS can also identify multiple fungal species simultaneously, which is important in cases of polymicrobial infections. By sequencing specific genes, NGS can detect genetic markers associated with antifungal resistance, guiding clinicians in selecting the most effective treatments. This capability is essential for optimizing treatment outcomes and reducing the risk of antimicrobial resistance. Moreover, NGS significantly reduces the time required for etiological diagnosis and aids in the selection of the most effective antimicrobial agents by pinpointing specific bacterial pathogens. The utility of NGS extends to pandemic pathogens such as tuberculosis and COVID-19 (37, 38). In the context of tuberculosis, NGS can rapidly identify Mycobacterium tuberculosis and its drug resistance profile, facilitating timely and targeted treatment (37). Similarly, during the COVID-19 pandemic, NGS has been instrumental in tracking viral mutations and understanding transmission dynamics (38). Finally, NGS helps researchers understand the dynamics of fungal infections, discover new drug targets, and study the fungal microbiome across various body sites. These applications highlight the versatility and importance of NGS in modern infectious disease management.

Recent studies (39, 40) have suggested that viruses play a crucial role in pneumonia, a notion that contrasts with traditional thinking that often downplays the significance of viral causative agents. The current study did not statistically analyze this aspect, but it did note that several patients received antiviral therapy, indicating a recognition of the potential role of viruses in pneumonia (30). Furthermore, a recent study found that patients with viral-bacterial co-infections experienced more severe conditions, including haemodynamic disturbances, respiratory failure, and more frequent hospitalizations compared to those with bacterial infections alone (41). These findings suggest that viruses may play a more critical role in some patients with sepsis than previously thought. In conclusion, the use of NGS as a complementary diagnostic tool is essential for the detection of a wide range of pathogens, including viruses, which can help guide optimal treatment strategies for patients with pneumonia and sepsis. The maturity of the technology, its improvement and wide clinical application, as well as its advantages in pathogen identification are clearly demonstrated by this study.

The study's findings underscored the value of NGS as a diagnostic tool in the context of complex pathogen identification, with an AUC of 0.884. These results highlighted NGS as an essential adjunct in diagnosing complex infections, where traditional methods often fell short. However, the relatively lower specificity of NGS necessitated careful interpretation of results to prevent the misuse of antibiotics and to accurately distinguish between pathogenic and non-pathogenic microorganisms. The criteria established for positive NGS results in this study have reached a consensus, but they do not definitively distinguish between pathogenic, background, and cell-free DNA from non-pathogenic microorganisms. Consequently, the fourth item on the criteria list becomes particularly crucial. The study found that NGS typically identified one or two pathogens per sample, with no more than six pathogens detected in a single instance, which suggested a targeted approach to pathogen identification. The AUC of NGS was 0.884, the combination of ALB and NGS significantly improved the diagnostic efficiency, with an AUC of 0.972, this combination served as a vital adjunct in reducing the risk of underdiagnosis and misdiagnosis, enhancing the precision of clinical assessments.

Pneumonia, a clinical syndrome arising from the immune system's response to infection, benefits from a thorough understanding of its etiology. Identifying at-risk patients and implementing preventive measures are key to managing pneumonia. The use of NGS on nasopharyngeal samples in pneumonia patients improves the detection sensitivity for rare and fastidious bacteria, particularly when combined with ALB. Despite the promise of NGS in detecting mixed infections, the absence of standardized diagnostic criteria means that the interpretation of NGS results must be integrated with clinical, laboratory, and physical examination findings. This integrated approach is particularly critical for patients with mild pneumonia, who may not present with classic symptoms or signs.

The study acknowledges several limitations, including its focus on pediatric patients and the predominance of nasal swab samples, which may not be representative of a broader spectrum of clinical infectious diseases. The interpretation of sequencing results lacks universally accepted standards, relying on clinicians' subjective judgment influenced by their experience. The study's reliance on three chief physicians for result interpretation introduces potential bias, despite their expertise. Financial considerations and health insurance coverage may have influenced patient selection, potentially introducing selection bias. The high cost of NGS testing may also limit its accessibility for patients with limited resources. Strict aseptic techniques during testing are essential to prevent clinical misdiagnosis due to the method's high sensitivity. The findings from this single-center study with a relatively small sample size must be validated in larger, multicenter clinical trials to confirm the results and establish more robust guidelines for the clinical application of NGS in diagnosing pneumonia. Future advancements in diagnostic methodologies may pave the way for more accurate and effective strategies in the diagnosis and treatment of pneumonia. Notably, the retrospective design and dataset limitations of this study precluded prevented detailed quantification and adjustment for prior antibiotic exposure. Future research should address this to strengthen findings. Moreover, we used NGS to identify viral and bacterial pathogens in clinical samples. while this study did not specifically focus on fungal pathogens, we recognize their potential importance, especially among vulnerable populations, such as children. Future research should take into account the inclusion of fungal pathogens to offer a more comprehensive evaluation of pathogen diversity. Additionally, the RMP ≥ 25 threshold, based on a single negative control, limits generalizability. Future studies should validate this threshold with more controls and conduct sensitivity analyses with different cut-offs. The study also did not assess intra- or inter-run reproducibility or limit-of-detection, which are crucial for diagnostic assay reliability. Future research should include these assessments to enhance robustness.

## Conclusions

NGS is a powerful diagnostic tool for identifying the etiology of pediatric pneumonia with speed and accuracy. Combining NGS with albumin levels could serve as an effective screening strategy, potentially improving clinical outcomes through earlier intervention and targeted therapies. However, the clinical utility of this approach requires further validation through larger cohort studies to fully realize its potential.

## Data Availability

The original contributions presented in the study are publicly available. This data can be found here: NCBI Sequence Read Archive (SRA), https://www.ncbi.nlm.nih.gov/sra/PRJNA1398601, Accession to cite for these SRA data: PRJNA1398601.
